# Sequence characteristics of T4-like bacteriophage IME08 benome termini revealed by high throughput sequencing

**DOI:** 10.1186/1743-422X-8-194

**Published:** 2011-04-27

**Authors:** Xiaofang Jiang, Huanhuan Jiang, Cun Li, Sheng Wang, Zhiqiang Mi, Xiaoping An, Jiankui Chen, Yigang Tong

**Affiliations:** 1 State Key Laboratory of Pathogen and Biosecurity, Beijing Institute of Microbiology and Epidemiology, Beijing 100071, China; 2 Clinical Laboratory, No.307 Hospital, Beijing 100071, China

**Keywords:** T4-like bacteriophage, terminase, high throughput sequencing

## Abstract

**Background:**

T4 phage is a model species that has contributed broadly to our understanding of molecular biology. T4 DNA replication and packaging share various mechanisms with human double-stranded DNA viruses such as herpes virus. The literature indicates that T4-like phage genomes have permuted terminal sequences, and are generated by a DNA terminase in a sequence-independent manner;

**Methods:**

genomic DNA of T4-like bacteriophage IME08 was subjected to high throughput sequencing, and the read sequences with extraordinarily high occurrences were analyzed;

**Results:**

we demonstrate that both the 5' and 3' termini of the IME08 genome starts with base G or A. The presence of a consensus sequence TTGGA|G around the breakpoint of the high frequency read sequences suggests that the terminase cuts the branched pre-genome in a sequence-preferred manner. Our analysis also shows that terminal cleavage is asymmetric, with one end cut at a consensus sequence, and the other end generated randomly. The sequence-preferred cleavage may produce sticky-ends, but with each end being packaged with different efficiencies;

**Conclusions:**

this study illustrates how high throughput sequencing can be used to probe replication and packaging mechanisms in bacteriophages and/or viruses.

## Background

T4-like bacteriophages share several characteristics with other double-stranded viruses, including lambda-like phages and herpes viruses[1-3]. One striking feature is the DNA replication and packaging mechanisms that involve the coordinated actions of numerous proteins[4-6]. There is evidence that T4 phage DNA replication and recombination processes generate a highly branched concatemeric DNA, which is then cut and packaged into an empty protein shell (prehead)[2,7]. Terminases (gp16 and gp17) are thought to carry out the digestion, whereby gp16 recognizes a DNA substrate and directs the larger gp17 subunit to the cleavage site, which is then cleaved by gp17 associated endonucleolytic activity[8,9].

Two general packaging mechanisms have been described for double-stranded DNA bacteriophages[10]. In phage lambda, T7 and T3, DNA ends with unique sequences are generated by terminases that recognize and cleave the *cos *sites[11]. While in phage P22, SPP1 and P1, the pre-genome DNA is cleaved in a strictly headful mechanism, in which DNA packaging starts in the vicinity of a packaging site, *pac*, and terminates at a length a little longer than the genome, with both termini generated by sequence-independent cleavage. The mechanism of T4-like phage DNA packaging remains obscure. The T4-like phage genome does not contain a *cos *site. Although the T4-like phage genome is also packaged in a headful mode, the terminal ends appear to be generated at random sequences across the genome[12,13].

High throughput sequencing is a novel tool for molecular biology studies and has found application in a variety of fields. It can serve as a powerful tool for in-depth genome sequence and gene expression analysis. We have isolated a novel *Enterobacteria *bacteriophage, IME08, from local hospital sewage[14]. Preliminary study with Sanger sequencing of random PCR clones revealed that IME08 was a T4-like phage. Since the T4-like bacteriophages have a genome of 150 to 230 kilobases, we adopted a high throughput sequencing strategy to sequence its genome. Following genome sequence assembly and gene annotation, we observed several interesting characteristics of the genome termini that would have never been revealed using conventional techniques. Here we demonstrate with high throughput sequencing that the termini of IME08 carries consensus sequences, indicating that it may adopt a mechanism of sequence-preferred cleavage, contrary to previous assertions. This study also demonstrates the use of high throughput sequencing techniques to study virus DNA replication and packaging.

## Methods

### DNA sequencing and sequence assembly

Phage IME08 genomic DNA was extracted as previously described[14], and sequenced using the Solexa Genome Analyser (Illumina, San Diego, CA, USA) at BGI (formerly known as the Beijing Genomics Institute). The sample preparation, library construction and sequencing by synthesis were performed according to Illumina's paired end sequencing protocols. Briefly, the phage IME08 genomic DNA sample was sheared to about 500 bp using a compressed air device nebulizer. After the ends of the sheared DNA were blunted using Illumina's Blunting Enzyme Mix, an A base was added to the 3' termini to generate 3' protrude double-stranded DNA molecules. A Y structure adapter (formed by Oligo1: 5' ACA CTC TTT CCC TAC ACG ACG CTC TTC CGA TCT 3', and Oligo2: 5' p-GAT CGG AAG AGC GGT TCA GCA GGA ATG CCG AG 3') was ligated to the 3' protrude DNA fragments. The ligated fragments of about 550 bp were isolated via gel extraction and amplified by 15 cycles of PCR using the primer PE1 (5' CAA GCA GAA GAC GGC ATA CGA GAT CGG TCT CGG CAT TCC TGC TGA ACC GCT CTT CCG ATC T 3') and PE2 (5' AAT GAT ACG GCG ACC ACC GAG ATC TAC ACT CTT TCC CTA CAC GAC GCT CTT CCG ATC T) to generate sequences with different adaptor sequences. The PCR products were loaded onto the flowcell of Illumina Solexa Genome Analyser machine, where the DNA molecules hybridized with the flowcell bound single stranded oligonucleotides complementary to the sequences of the abovementioned PCR primers. After PCR-based "cluster generation" by "bridge amplification" [15], "sequencing by synthesis" was performed sequentially using read 1 sequencing primer (5' TGT GAG AAA GGG ATG TGC TGC GAG AAG GCT AGA 3') and read 2 sequencing primer (5' CGG TCT CGG CAT TCC TGC TGA ACC GCT CTT CCG ATC T 3') to generate two raw sequencing read files (1.fq and 2.fq) with read length of 73 bp and 75 bp respectively. The complete genomic sequence of phage IME08 was then assembled by Velvet[16] and verified with other assembly software including ABYSS[17] and SOAPdenovo[18].

### Bioinformatics analysis

The potential coding regions of the IME08 genome was predicted using the software Kodon (Applied Math, Sint-Martens-Latem, Belgium) with a minimum open reading frame (ORF) size of 50 amino acids, and with the "Bacterial and Plant Plastid Code" as translation table. The putative coding regions were then BLASTed against the bacteriophage genome database downloaded from the European Molecular Biology Laboratory (EMBL). The best matches were used to annotate the IME08 ORFs. A total of 253 ORFs were identified and annotated. tRNA genes were predicted using tRNAscan-SE (v.1.21)[19].

### Nucleotide sequence accession number

IME08 sequence data were deposited at GenBank under the accession number NC_014260.

## Results and Discussion

### High throughput sequencing, contig assembly and gene annotation

High throughput sequencing of the IME08[14] genome by the Solexa Genome Analyser generated 5,011,480 pairs of reads (73 bp and 75 bp, respectively), which is about 742 Mbp, or 4,300-fold coverage. To assemble the genomic sequence using Velvet, we first extracted a small fraction of paired-end data (about 20-fold coverage) from the original data files to test the k-mer value, and determined that k-mers of 27 to 31 gave optimal results. Different amount of paired-end data were tested to assemble the IME08 genome and the results showed that 20-100 fold coverages (random subset data) were capable of assembling the full-length genome sequence as a single contig, without any gaps or unresolved nucleotides.

The assembled full-length contigs varied in length and contained redundant duplicated sequences at the ends, suggesting a circular genome. This is consistent with the characteristic T4-like phage genome, which is circularly permuted and terminally redundant[20]. The accuracy of the Velvet[16] assembly was further verified by assembly tools ABYSS[17] and SOAPdenovo[18], and by aligning the assembled sequence with homologous T4-like phage genomes.

The genome of IME08 consists of 172253 bp, with an average GC Content of 39%. About 90% of the genome encodes a total of 253 predicted protein genes (CDSs) and three tRNA genes. Homology analysis indicated that IME08 is closely related with T4-like phage JS98[21,22]. Detailed genomic analysis and evolutionary study of IME08 is in preparation.

### High frequency read sequences suggest that T4-like phage genome termini are located at hot-spots

**Figure 1:**
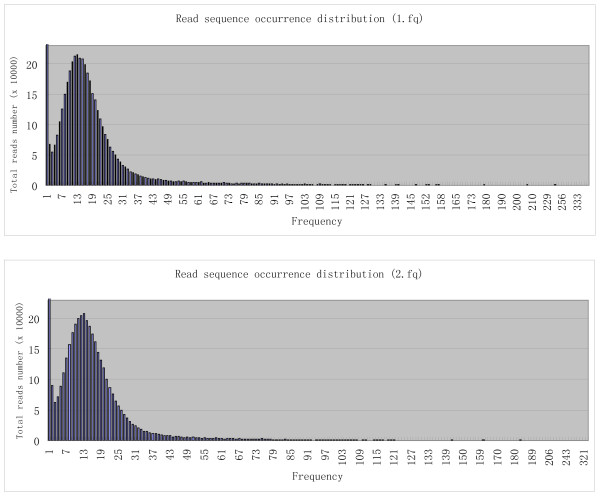
**Read sequence distribution**. The occurrence frequencies of sequences in raw data file 1.fq (shown in the upper graph) and 2.fq (shown in the lower graph) show that although the majority of the read sequences have 6-21 occurrences, high occurrence reads exist in both raw read files.

**Figure 2:**
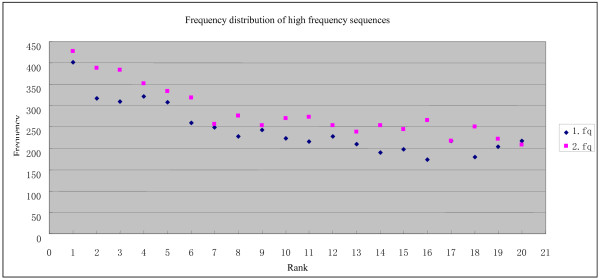
**Frequency distribution of the top 20 high frequency sequences (HFSs) in the original sequencing data files**.

It may be possible to find which reads are located at the genome termini, given the large volume of sequence data. If genome termini are generated randomly, there should not be a very high frequency of individual read sequences. To determine sequence frequency, raw read files (1.fq and 2.fq) were sorted by sequence and the occurrence of each unique sequence was calculated. Frequency statistics (Figure 1) demonstrated that most sequences in both raw files have 6-22 occurrences, with the most frequent occurrence at 13. The read sequences that occurred 6-22 times comprise about 70% of all sequences, excluding single occurrences. Single read sequences probably contain many sequences derived from quasi-species (with single nucleotide polymorphisms, SNPs), or from sequencing errors (with one or more base-calling errors). In contrast, although the average occurrence is 13, high frequency sequences (HFSs) had up to 400 occurrences in a single raw sequencing file (Table 1 and Figure 2). Further analysis showed that the HFSs in 1.fq and 2.fq read files contain identical sequences. These sequences are unique since they occur only once in the assembled genome. BLAST analysis revealed that these sequences have homology with unique sequences of other evolutionally related T4-like bacteriophages (T4, JS98 and JS10). There is no evidence of endogenous plasmid contamination in the raw sequencing data, making it unlikely that these HFSs arise from contaminated multi-copy plasmids. Further analysis demonstrated that the paired end sequences of these HFSs occurred at normal frequencies (data not shown), indicating that they were not produced by PCR amplification prior to cluster generation during the Solexa sequencing process. The read sequences (forward) within a few bases upstream or downstream of the HFSs in the genome occurred at normal frequencies, which again suggests that the HFS reads were not sequence-independently generated from particular vicinities.

**Table 1:** Top 20 high frequency sequences in raw sequencing data

read sequence	frequencies				genome presence		
	
	rank	total	1.fq	2.fq	Ori^1^	Pos^2^	Junction^3^
GCTCTTCGGAAAGGTCAAAAACAGTTTGAG......	1	828	427	401	F	30641	tctatttggagctcttcgga

GTTTTACAGAATCGTACTCGGCCTTGTTCG......	2	705	388	317	F	3272	aattactggagttttacaga

GTATAATGATTCATCAACAAACAAAAGACA......	3	692	383	309	R	30486	cccttttggagtataatgat

GCGTAATTCCACCTTTTTCTTCCCAATCTT......	4	673	352	321	F	52555	tcttgttggagcgtaattcc

GGTATACATCATTAAATAACGATGTATATC......	5	641	333	308	F	163251	agaaattggaggtatacatc

GTATTTCAAGAAACGTGATAAAGCCCAGGC......	6	577	318	259	F	121764	aacgtttggagtatttcaag

GCGTAATTGCTTCAGGTAAGCCTTTAGGAT......	7	505	256	249	F	73140	agaatatggagcgtaattgc

GTGCATGATTGGTAACAGTTCGGCAACCCA......	8	505	277	228	F	40411	ggtctttggagtgcatgatt

GTTTTACAGACAACGCAAATCTTATCTGAC......	9	496	253	243	F	115803	atcgattggagttttacaga

GCTGAAAAGGCAGCTGAAACTAAAGCCGCT......	10	494	270	224	R	3702	taaattagcagctgaaaagg

GTATAATGTAAAAACAAACCTGAGGAAATT......	11	490	274	216	R	32654	ctcccttggagtataatgta
GTATTAACAAGATTCCAGAATTTCTCACCC......	12	481	253	228	R	75276	gttttctggagtattaacaa

GTTTCTCAGCGATTTTAATCGACCACTCTT......	13	448	238	210	F	29924	tcgtcttggagtttctcagc

GTTACATAAGCATCAGGAGCAGATGGTCCC......	14	445	254	191	R	102003	ttgctttggagttacataag

GCTTTAATCTTAACAATAGTGCCGAGATAA......	15	443	245	198	F	165136	gtatttacctgctttaatct

GCTGAACGTACCGAAGTTGCAGGTATGACT......	16	440	266	174	R	28799	gttgttcagagctgaacgta

GTATAATCTTTCTATCAACTTGAGGAGAAT......	17	434	217	217	R	46215	gatggatggagtataatctt

GCTGCATCTTCAGATTGGTCTTCGTCTTCA......	18	431	251	180	F	5448	ttcagatggagctgcatctt

GTTATTACTAAACAAGTTTTTAACCGCACT......	19	426	222	204	R	122567	ctcccttggagttattacta

GTTAACAAATGCCATACGACATTTAAGGGA......	20	425	208	217	F	56968	aacgtttagagttaacaaat

^1 ^Orientation in genome. F, forward; R, reverse.

^2 ^Position in genome.

^3 ^The genomic sequence around the start site of the HFSs.

The phage IME08 genome is relatively small compared with the genomes of cellular organisms (such as prokaryotic or eukaryotic species). Since no obvious repeat sequences were observed, the highly elevated occurrence of particular unique sequences suggests they are not randomly sheared at library construction, but are the original terminal sequences which already exist prior to shearing in large amount in the phage genomic DNA sample. This result indicates that the termini of the T4-like phage IME08 genome were generated by terminase cleavage at particular hot spots.

### T4-like phage genomes start with G or A bases

**Figure 3:**
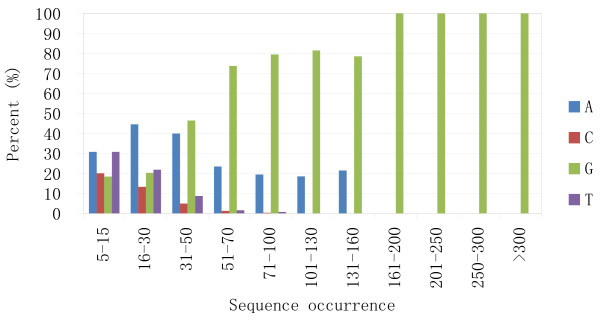
**Percentage of first bases in read sequences**. In read sequences with 5-15 occurrences, the first base percentage is comparable with the base composition in the genome. As the read sequence occurrence increases, the percentage of A and G goes up and comprises 100% of the first bases in sequences that occur more than 100 times. Base G is the only first base in sequences that occur greater than 160 times.

When the first bases of the read sequences of all occurrences were plotted (Figure 3), a striking characteristic was revealed. The first bases of HSFs were dominated by A and G, and as sequence occurrence increased, G exceeded A as the major start base. G was the sole base for all sequences that occurred more than 160 times (Figure 3). The first base plot also revealed that for sequences that occurred more than 30 times, A and G comprised more than 80% of the first bases, with T or C making up less than 10% (percentage of C is fewer than that of T because the GC content of IME08 phage genome is only 39%). For sequences that occurred more than 100 times, there is never a T or C base located at the first base. This distinct difference in the first bases between high frequency sequences and normal frequency sequences again suggests that HFSs are different from the normal frequency sequences which are supposed to be generated randomly at library construction, and that these HFSs may represent the termini of the original genomic DNA. According to this hypothesis, G should compose the majority of the genome terminal bases since it is the only 5' terminal base in the HFSs with very high frequencies.

### Consensus sequences of HFSs reveal sequence-preferred cleavage by T4-like phage terminase

**Figure 4:**
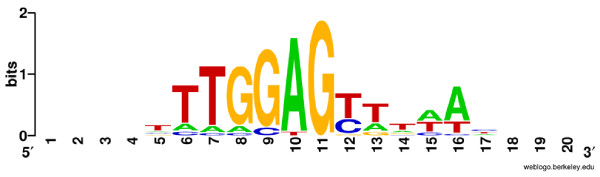
**Sequence logo representation of sequences around the start sites of the top 20 HFSs**. Sequences were plotted with WebLogo [23]. The height of the letter indicates the degree of conservation. Nucleotide 11 is the start site of HFSs.

To characterize HFSs, various analysis measures were applied, including consensus sequence searches, homology searches, and local thermal stability (melting temperature) plotting. For consensus sequence analysis, the top 20 HFSs together with their upstream sequences (retrieved from the assembled genome) were plotted with Weblogo[23]. The results demonstrate an obvious consensus sequence around the cleavage breakpoint, with the major part of the consensus sequence not included in the HFS sequences, but in its upstream sequences (Figure 4). Of the top 20 HFSs, 16 (80%) have an identical cleavage site 5'-TTGGA↓G-3', indicating cleavage is highly sequence-preferred (not strictly sequence-specific). Since the forward and the reverse HFS sequences (relative to the genome orientation) have the same consensus cleavage sequence, it suggests that both 5' and 3' genome terminus cleavage share a common mechanism, which indicates that the packaging of genomic DNA is bidirectional (but the two ends of a particular genome are not cleaved with the same mechanism. The first cleavage is highly sequence-preferred and the second cleavage is probably random or nearly random, as discussed later in this paper). To extend the search for other sequence characteristics around the cleavage sites, the -100 to +100 nucleotides of the HFS cleavage sites were analyzed with Weblogo and Clustal software. The results did not reveal any additional consensus sequences (data not shown).

All the above analyses demonstrate that the mechanism for generating the T4-like phage IME08 termini involves a highly sequence-preferred cleavage. This is inconsistent with previous reports by Bhattacharyya *et al *that T4 terminase (gp16/gp17) cleaves genomic DNA in a sequence-independent manner[12,13]. The most possible explanation for this inconsistency may be that the terminase is only sequence-preferred, but not strictly sequence-specific, and it can cut both in a sequence-preferred manner (the first cut of a particular genome) and in a nearly random manner (the second cut of a particular genome, see discussion below). When the terminase was expressed from a strong promoter like phage T7 or λ pL promoters which was used by Bhattacharyya *et al*, the terminase was produced in large quantity and led to excessive random digestion of the plasmid DNA and the host bacterial chromosome DNA[12,13]. Since the plasmid was too small and might not contain any sequence suitable for sequence-preferred digestion, and the host bacterial DNA was too large and contained large amount of terminase preferred sequences, the resultant cleavage product was a mixture of DNA molecules with both consensus sequence termini and nearly random sequence termini. These termini can not be characterized be conversional sequencing techniques due to the large amount of random terminal sequences. In this context, high throughput sequencing is the best tool for the study.

### Unbalanced occurrence of forward and reverse HFSs indicate packaging of the genome was asymmetric and that 5' termini are less permuted than 3' termini

To further characterize HFSs, the top 20 HFSs (Table 1) as well as the top 200 HFSs (additional file 1) were analyzed for their orientation relative to the genome. This analysis showed that the forward HFSs dominate in the two raw sequencing data files, with both having a forward vs. reverse ratio of roughly 1.5:1. Among the top 10 HFSs there was only one reverse sequence (Table 1). The higher occurrence of forward HFSs suggests that the 5' terminus (relative to genome orientation) were less permuted than the 3' terminus. This phenomenon also indicates that the genomic DNA packaging is asymmetric. This difference may be explained by either of the following mechanisms. The first mechanism is that recombination-dependent DNA replication is asymmetric[24], and forward replication dominates the process, resulting in more genomes with forward HFSs being generated and packaged. Alternatively, it may be that the forward sequence contained some sequences more preferred by the terminase.

### Sequence-preferred cleavage may produce sticky-ends and both ends may be packaged but with different efficiency

**Figure 5:**
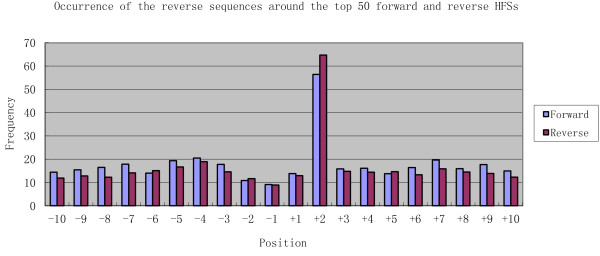
**Occurrence of the reverse sequences around the top 50 forward and reverse HFSs**. The frequencies of the reverse sequences starting from a particular position around the first base of the HFSs were calculated. The frequency profile of the forward and reverse HFSs are near identical, with position +2 reverse sequences (R+2) having a remarkablely higher occurrence than the others.

**Figure 6:**
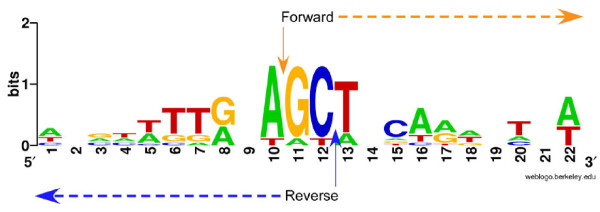
**Sequence logo representation of sequence around the start sites of paired HFSs**. Sequences were plotted with WebLogo [23]. Height of letter indicates degree of conservation. Nucleotide 11 is the start site of forward HFSs.

To test if both ends generated from the same terminase cut can be simultaneously packaged into two viral heads, occurrence of the reverse sequences located around the HFSs were counted. The results highlighted an interesting phenomenon. For most of the HFSs, the reverse sequences starting from position +2 (R+2) occurred at a striking higher frequency than those from any other position (Figure 5 and additional file 2). The high frequency of these R+2 sequences indicates that such sequences should also be the genome termini, suggesting that both ends of a single terminase cleavage could be packaged. Consensus sequence analysis showed that these paired HFSs contained a core consensus sequence TTGNA|GCT (Figure 6) which is slightly different from the above defined top HFS consensus sequence. The forward and reverse HFS pairs have an overlap of two base pairs, indicating terminase cleavage probably generated sticky ends with a 2 nucleotide 5' overhang (mostly GC) (Figure 6). Although it has been shown that T4 genomic DNA can be directly ligated[25], it does not necessarily mean that the termini are blunt-ended, since sticky ends can be ligated with high efficiency.

Since the forwards HFSs and their reverse counterparts R+2 occur at different frequencies (some of these differences are very large), the two ends generated by the same cleavage may not be simultenously packaged into two virions (i.e., one packaged, the other lost), and there may be a packaging preference between the two ends derived from the same cleavage.

### Unpaired distribution of HFSs on the genome suggests distinct processes for upstream and downstream genome ends, with one end generated in a sequence-preferred manner and the other randomly generated

**Figure 7:**
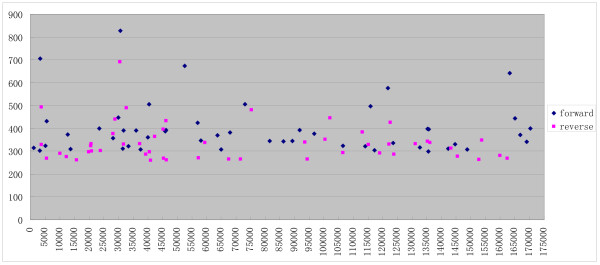
**Distribution of top 50 forward and reverse HFSs on the IME08 genome**.

It is assumed that both ends of T4-like phage genomes are processed by the terminase. Our results suggest that both the forward and the reverse termini have similar terminal consensus sequences. Since T4-like phage have a fixed genome length (though circularly permutated), which is about 102% of its genome size[20], if both ends of a genome are flanked by HFS sites, then HFS sites should exist as pairs along the genome with the forward HFS sites located about 2% of the genome (about 3.4 kb) upstream of the reverse HFS sites. However, genome distribution analysis of the HFSs did not reveal a position correlation between the forward and the reverse HFSs (Figure 7 and additional file 3). In some regions, HFSs of only one orientation were observed (e.g. only forward HFSs exist in regions 70-90k, 164-2k, and only reverse HFSs exist in 150-164k). The unpaired distribution pattern thus contradicts the assumption that both ends of a genome are generated with the same cleavage mechanism, and suggests that for a particular genome, one end is generated by a sequence-preferred mechanism, whereas the other end is produced randomly, with a headful size-dependent cleavage. In this hypothetic packaging mechanism, the terminase will first recognize and cleave the consensus sequence, and insert one end into the virion prehead. The package process continues until a headful length of genomic DNA is loaded into the prehead when the terminase executes a second cleavage to terminate the DNA package process. This second cleavage of the genome may be less sequence-preferred or even random.

Genome distribution of the top 50 forward and top 50 reverse HFSs also indicates that the HFSs are not evenly distributed alone the genome, which may reflect the activities of genome replication initiation or transcription. The overall frequencies of forward HFSs are greater than that of the reserve HFSs (Figure 7 and additional file 3), again suggesting that the replication and packaging of the genome is asymmetric and that the forward ends (5' relative to the genome) are more permuted than the reverse ends (3' relative to the genome).

## Competing interests

The authors declare that they have no competing interests.

## Authors' contributions

XJ, HJ and CL conducted IME08 sequence assembly, gene annotation and drafted the manuscript. SW, ZM and XA participated in preparation of the genomic DNA. YT and JC conducted the design of the study and writing of the manuscript. All authors read and approved the final manuscript.
